# Harnessing the Effects of BTKi on T Cells for Effective Immunotherapy against CLL

**DOI:** 10.3390/ijms21010068

**Published:** 2019-12-20

**Authors:** Maissa Mhibik, Adrian Wiestner, Clare Sun

**Affiliations:** Laboratory of Lymphoid Malignancies, Hematology Branch, NHLBI, NIH, Bethesda, MD 20892, USA; maissa.mhibik@nih.gov (M.M.); wiestnea@nhlbi.nih.gov (A.W.)

**Keywords:** chronic lymphocytic leukemia, microenvironment, T-cell, Bruton tyrosine kinase inhibitors, immunotherapy, combination strategies

## Abstract

B-cell receptor (BCR) signaling and tumor–microenvironment crosstalk both drive chronic lymphocytic leukemia (CLL) pathogenesis. Within the microenvironment, tumor cells shape the T-cell compartment, which in turn supports tumor growth and survival. Targeting BCR signaling using Bruton tyrosine kinase inhibitors (BTKi) has become a highly successful treatment modality for CLL. Ibrutinib, the first-in-class BTKi, also inhibits Tec family kinases such as interleukin-2–inducible kinase (ITK), a proximal member of the T-cell receptor signaling cascade. It is increasingly recognized that ibrutinib modulates the T-cell compartment of patients with CLL. Understanding these T-cell changes is important for immunotherapy-based approaches aiming to increase the depth of response and to prevent or treat the emergence of resistant disease. Ibrutinib has been shown to improve T-cell function in CLL, resulting in the expansion of memory T cells, Th1 polarization, reduced expression of inhibitory receptors and improved immune synapse formation between T cells and CLL cells. Investigating the modulation of BTKi on the T-cell antitumoral function, and having a more complete understanding of changes in T cell behavior and function during treatment with BTKi therapy will inform the design of immunotherapy-based combination approaches and increase the efficacy of CLL therapy.

## 1. Introduction

Chronic lymphocytic leukemia (CLL) is a common B-cell malignancy characterized by the expansion of mature monoclonal B lymphocytes in the blood, bone marrow and lymphoid tissues. Interactions between tumor cells and their microenvironment trigger B-cell receptor (BCR) activation and support tumor growth and survival [1]. Inhibition of BCR signaling has become a highly successful treatment strategy for CLL and other B-cell malignancies. Among the first approved BCR kinase inhibitors, ibrutinib inhibits Bruton tyrosine kinase (BTK), and has achieved high response rates and durable remissions in CLL patients [2]. However, complete responses are rare, and drug resistance due to mutations in BTK and/or Phospholipase C Gamma 2 (PLCG2) is an emerging clinical problem [3]. Therefore, adjunct treatment is needed to deepen response and to prevent or overcome drug resistance.

Ibrutinib, whether directly through the inhibition of kinases other than BTK or indirectly through suppression of tumor microenvironment cross-talk, affects immune cells, of which T cells have been the most studied [4].

Within the microenvironment, T cells contribute to the maintenance of tumor cells. T cells provide pro-survival signals through soluble factors such as interleukin-4 (IL-4) and interferon-gamma (IFN- γ), which upregulate anti-apoptotic Bcl-2 in CLL cells, [5,6] and by direct interactions via CD40L-CD40 [7]. In a the patient-derived xenograft model, co-infusion of autologous CD4^+^ T cells is required for the engraftment and clonal expansion of CLL cells, indicating their critical role in leukemogenesis [8]. In addition, abnormal T-cell subset distribution and function result in the failure of antitumor immunity [9].

Evaluation of the T-cell compartment may yield critical insights into the mechanism and limitations of current therapies. Several studies have shown the immunomodulatory effects of ibrutinib. In this review, we discuss the effect of ibrutinib on T cells and the potential of harnessing these changes to improve disease control by combining ibrutinib with immunotherapy.

## 2. Improved Antitumor T-Cell Responses during Treatment with Ibrutinib

Besides BTK, ibrutinib inhibits other kinases from the Tec family including the interleukin-2-inducible T-cell kinase (ITK) expressed by T cells [10]. Although off-target kinase inhibition by ibrutinib may account for some adverse effects, such as diarrhea, rash, atrial fibrillation and bruising [11], it has been hypothesized to improve T-cell immunity [10].

### 2.1. Absolute Number of T Cells

Patients with untreated CLL show an increase in the absolute number of T lymphocytes compared to age-matched healthy donors, relative expansion of CD8^+^ T cells in circulation, and inversion of the normal CD4:CD8 ratio [12,13,14]. An inverted CD4:CD8 ratio has been associated with more advanced disease and shorter time to first treatment [14,15]. Patients with baseline T lymphocytosis showed a decrease of T-cell counts into the normal range by 6 to 12 months from the start of their ibrutinib therapy [16,17,18]. In contrast, Long et al. reported an increase in CD4 and CD8 T cells during the first six cycles of therapy in ibrutinib-treated patients [19].

### 2.2. T-Cell Receptor Repertoire

During T-cell development, unique variable domains of the α and β polypeptide chains are generated via somatic recombination of the V, D and J gene segments. Recognition of peptide antigen by the α/β heterodimeric T-cell receptor (TCR) leads to a clonal expansion of T cells containing the same hypervariable complementarity determining region 3 (CDR3). CDR3, in particular, specifically recognizes antigen presented by a major histocompatibility complex (MHC) molecule.

The first evidence of T-cell oligoclonal expansion in CLL was identified by Southern blot in 1990 [20]. Restriction of TRBV gene usage was confirmed by flow cytometry and spectratyping approaches. [21,22,23]. A skewed TCR repertoire occurs early in the disease course, even among individuals with the CLL precursor condition monoclonal B-cell lymphocytosis (MBL). T-cell clonal expansion parallels the numerical increase of clonal B cells. Hence, it has been suggested that select T-cell clones expand in response to tumor-specific antigens [24]. In recent years, sequencing of the TRBV CDR3 region has been used to study the T-cell repertoire, diversity of TCR subfamilies and antigen-specific expansion of T-cell clones. Using low-throughput subcloning followed by Sanger sequencing, Vardi et al., identified T-cell repertoire skewing and TRBV gene restrictions that were shared by patients belonging to a shared CLL subset, defined by B-cell receptor (BCR) stereotypy [25]. A follow-up study using next-generation sequencing (NGS) in major stereotyped subsets showed the pronounced oligoclonality of CD8^+^ T cells with clones that persist and expand over time. Whether tumor neoantigens are implicated in the selection and expansion of T cells is not fully understood [26].

Next generation sequencing of the TRBV repertoire in refractory/relapsed (R/R) CLL patients revealed an increase in TCR diversity after 12 months of treatment with ibrutinib alone or in combination with rituximab. While dominant clones declined, the number of unique clones increased during treatment with ibrutinib. TCR repertoire diversity was positively correlated with clinical efficacy and inversely correlated with the rate of infections [17]. However, a separate study found a more clonal TCR repertoire at the time of response to single-agent ibrutinib, which was lost at disease progression [27]. A key difference between these two reports is the inclusion of treatment naïve patients in the latter analysis. Notably, T cell clonality was higher in relapsed/refractory patients than in treatment-naïve patients at the beginning of ibrutinib therapy. Differences in the size and composition of the baseline T-cell compartment could thus contribute to the discrepant findings.

### 2.3. Memory T Cells

**Figure 1:**
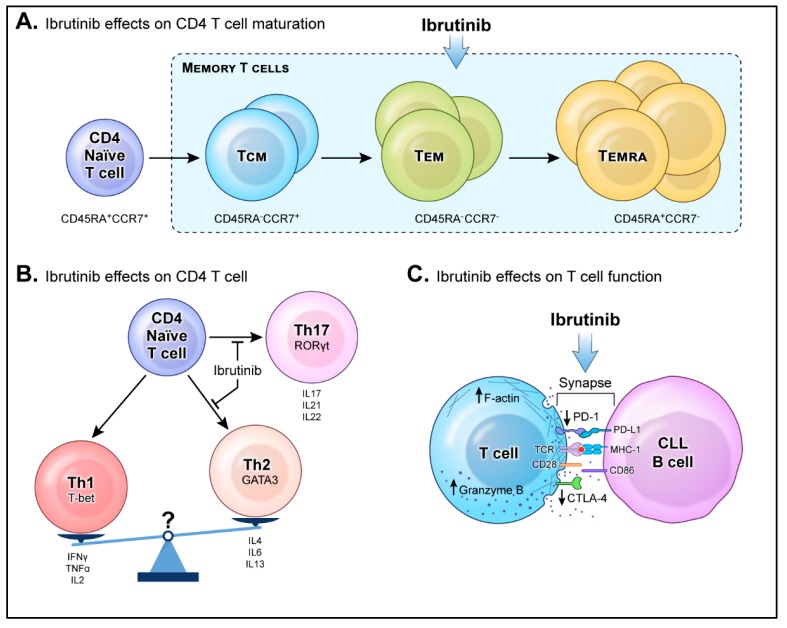
Ibrutinib shifts T-cell differentiation and function towards improved antitumor control, immunity and surveillance. This figure summarizes the reported effects of ibrutinib on T cells: (**A**) Ibrutinib affects the differentiation of naive CD4 T cells into memory T cells. After ibrutinib treatment, more differentiated memory T cells, including central memory (CM), effector memory (EM) and effector memory RA (EMRA), are increased; (**B**) Ibrutinib affects the differentiation of naive CD4 T cells into T helper cells shown by changes of different Th-mediated cytokines in serum or plasma of treated patients. Ibrutinib inhibits the polarization into Th17 and Th2 cells; the latter results in a shift towards Th1 and favors antitumor immunity; (**C**) Treatment with ibrutinib also repairs immune synapse formation between T cells and CLL cells by enhancing F-actin polarization and granzyme B production and decreasing the expression of inhibitory receptors such as PD-1 and CTLA4.

One of the challenges of cancer therapy is to promote the development of specific T cells that can effectively target tumor cells and provide long-term anti-tumor protective immune responses. Circulating memory T cells are composed of central memory (CM), effector memory (EM) and effector memory RA (EMRA) that can be defined by their pattern of expression of the lymph node homing receptors CCR7 and CD45RA [28,29]. A minor T-cell shift towards more differentiated CD45RA^−^CCR7^+^ CM and CD45RA^−^CCR7^−^ EM within the CD4^+^ subset was observed in untreated CLL compared to healthy individuals [15,30]. It has been proposed that the accumulation of memory T cells is driven by the chronic antigenic stimulation of naïve T cells by CLL cells within an inflammatory microenvironment [15,30,31]. After treatment with ibrutinib, more differentiated T cells such as EM and EMRA are further increased as shown in (Figure 1A) [19].

### 2.4. Th1 and Th2 Polarization

Anti-tumor immune surveillance is supported by a type 1 T-helper (Th1) cytokine response with the production of IL-2, IFN- γ, and TNF-α, and may be impaired by a type 2 T-helper (Th2) response with IL-4, IL-5 and IL-13 cytokine secretion [32]. Although T cells from CLL patients have defects in proliferation and cytotoxicity, they produce increased levels of TNF-α and IFN-γ [9]. Defining T-helper subsets by flow cytometry with the expression of chemokine receptors CCR6 and CXCR3, Palma et al., reported an increase of Th1, Th2, Th17 and regulatory T cells in CLL patients compared to healthy controls [31]. However, a shift from Th1 to Th2 polarization appears to predominate in CLL and stronger Th2 polarization has been linked to disease progression [33,34]. The balance between Th1 and Th2 polarization may be affected by ibrutinib treatment. Using in vitro and mouse models, Dubovsky et al. showed that Th2 differentiation depends on ITK, while Th1 cells express resting lymphocyte kinase (RLK), which serves a redundant role when ITK is inhibited. In a mouse model of leishmania infection, treatment with ibrutinib promoted a Th1-type inflammatory response with clearance of the parasite, while vehicle-treated mice succumbed to infection [10]. A shift towards the Th1 phenotype was also detected in healthy donor and CLL T cells treated with ibrutinib in vitro [10]. This study reported Th1 polarization in response to ibrutinib that was associated with T-bet upregulation and JunB downregulation [10].

In a more recent study, analysis of Th1 and Th2 transcription factors in CLL patients showed a reduction in GATA3-expressing Th2 cells and no change in T-bet-expressing Th1 cells after 3 to 12 months of ibrutinib treatment [35]. Multiple studies have investigated the effect of ibrutinib on Th1 and Th2 cytokines. In vitro treatment of CLL PBMCs with ibrutinib inhibited the production of inflammatory cytokines such as IL-6, IL-10 and TNF-α [36]. In vivo, Niemann et al. reported a decrease in the serum levels of multiple inflammatory cytokines and chemokines in ibrutinib-treated patients [16].

In plasma obtained from ibrutinib-treated patients, Th2 and Th1 cytokines decreased, while the Th1:Th2 ratio remained stable, after 12 months of treatment [17]. Although a decrease in Th2-type cytokines has been reported by several groups, a change in Th1-type cytokines has been less consistent [10,16,17,19,37].

Taken together, the potential shift in the Th1:Th2 balance during ibrutinib therapy is primarily mediated by suppression of Th2 differentiation rather than Th1 expansion. An integrated analysis combining immunophenotyping, transcription factors and cytokines may help to better define the effect of ibrutinib on Th1 and Th2 polarization (Figure 1B). Although the Th1/Th2 dichotomy may play an important role in CLL pathogenesis, more recent studies have described the key role of regulatory T-cells (Tregs) in limiting anti-tumor immune responses.

### 2.5. Th17 and Tregs Balance

The balance between intratumoral Tregs and Th17 is critical in the modulation of inflammation, immune response to pathogens and autoimmunity. In CLL, increased Tregs are associated with downregulation of Th17 cells and disease progression [38,39,40]. In particular, Th17 cells are downregulated by CD39^+^ Tregs [41,42]. CLL cells also contribute to this imbalance by expressing costimulatory molecules such as CD70 and CD80/86 and producing IL-10 or TGF-β, which promote the development of Tregs and suppress Th1, Th17 and cytotoxic T-cell responses [43,44]. However, the role of Th17 cells in CLL, particularly within lymphoid tissues, remains largely unknown.

ITK has been shown to regulate Th17 and Tregs differentiation. Ex vivo, under conditions favoring Th17 or Tregs differentiation, CD4^+^ T cells from ITK deficient (ITK^−/−^) mice were shown to differentiate more efficiently into Tregs and express more FOXP3 and less IL-17A than CD4^+^ T cells from wild-type mice. In vivo, CD4^+^ T cells also preferentially differentiate into Tregs in ITK^−/−^ mice [45]. Ibrutinib inhibits ITK, and was found to block ex vivo differentiation of healthy, naïve CD4^+^ T cells into Th17 [46]. FOXP3^+^Tregs expand in response to low doses of ibrutinib, but are inhibited at higher doses [46]. In patients receiving ibrutinib, a decrease of FOXP3^+^Tregs was observed during the first two months of therapy [19,37]. Further, decreases in Th17 cells (CXCR3^−^CCR6^+^) and Th17-mediated cytokines such as IL-17A, IL-21 and IL-23 were observed in the serum or plasma of CLL patients treated with ibrutinib [16,17]. Thus, ibrutinib modulates the Th17/Tregs balance by directly inhibiting ITK in T cells.

### 2.6. T-Cell Function

First described in chronic viral infections, exhaustion describes a state of reduced proliferative capacity and effector function of chronically (hyper-)stimulated T cells [47], and has now been observed across many cancer types [48]. In CLL, Riches et al. identified “pseudo-exhausted” T cells with reduced proliferative capacity and effector function, but preserved cytokine production [9]. In secondary lymphoid organs, T cell dysfunction is further exacerbated by [49] close interactions with CLL cells expressing high levels of inhibitory molecules [50].

CLL cells express inhibitory ligands that alter the expression of genes involved in cell differentiation and cytoskeletal formation in CD4^+^ and CD8^+^ T cells [50]. As a result, T cells, whether from CLL patients or healthy donors, lose their ability to form an immunological synapse when they are in direct contact with CLL cells [51]. Programmed cell death protein 1 (PD-1) is a widely studied inhibitory receptor. An increase in PD-1^+^ T cells has been described in CLL and other B-cell malignancies such as follicular lymphoma (FL), diffuse large B-cell lymphoma (DLBCL) and MCL [9,15,31,52]. Clinically, the presence and ongoing inhibitory effect of other immune checkpoint molecules may explain the lack of benefit of anti-PD-1/PD-L1 monotherapy in CLL [53,54]. In CLL, some of these additional immune checkpoints such as T-cell immunoglobulin domain and mucin domain protein 3 (TIM-3), lymphocyte-activation gene 3 (LAG-3), or T-cell immunoreceptor with Ig and ITIM domains (TIGIT) are coexpressed by PD-1^+^ T cells [55,56].

For example, the proportion of T cells co-expressing TIM-3 and PD-1 is higher in CLL than in healthy controls, and is associated with defective T-cell function and disease progression [56]. Targeting alternative or multiple immune checkpoints may represent a more effective strategy to reactivate T-cell immunity.

Ibrutinib has been shown to decrease the chronic activation of T cells in patients with CLL. Reduced expression of activation markers, CD39 and HLADR, and immune checkpoints, PD-1 and CTLA4, on T cells was observed after ibrutinib treatment [16,19,57]. These changes may be mediated by the downregulation of immunosuppressive factors, such as IL-10 or CD200, in CLL cells upon ibrutinib treatment [19,57]. Papazoglou et al. compared the effect of ibrutinib, in combination with rituximab to traditional chemoimmunotherapy on the T-cell function. In ex vivo cytotoxicity assays, T-cell killing of autologous CLL-cells improved in patient samples obtained after ibrutinib therapy compared to baseline. Treatment with ibrutinib also repaired immune synapse formation between T cells and CLL cells by enhancing F-actin polarization, tyrosine phosphorylation of proteins and granzyme B production, as summarized in (Figure 1C) [58].

### 2.7. Second Generation BTKi

The contribution of ITK inhibition by ibrutinib to changes in the T-cell compartment has been informed by studies of highly selective second generation BTKi. Acalabrutinib, a second generation irreversible BTKi, is highly selective for BTK compared with other kinases, such as such as EGFR, TEC and ITK (IC50 >1000nM) [59]. In contrast to ibrutinib, acalabrutinib showed less inhibition of LCK, SRC kinases and ITK phosphorylation within healthy T cells [60]. No significant changes in T-cell numbers were observed during the first 20 weeks of acalabrutinib treatment [19]. With extended treatment, CD8^+^ T-cell counts eventually decreased after 15 cycles of acalabrutinib [59]. Similarly, our clinical data showed a progressive decrease in CD4^+^ and CD8^+^ T-cell numbers starting at six months of treatment with acalabrutinib [61]. Treatment with acalabrutinib and zanubrutinib, another second generation BTKi, led to downregulation of PD-1 and CTLA-4 [19,62]. However, T helper subsets, specifically Th1/Th2 polarization, were unaffected [19,62]. These data suggest that although BTK inhibition alone may normalize T-cell counts and reverse the exhausted state of T cells, ITK inhibition might play a role in changing T-cell polarization during ibrutinib therapy. Interestingly, a recent report described BTK expression in T cells, especially in effector/memory T cells, and its potential role in T-cell activation, suggesting a direct on-target, off-tumor effect of BTKis on T cells [63].

## 3. Improved Efficacy with Combinations of BTKi and T-Cell Directed Immunotherapy

**Figure 2:**
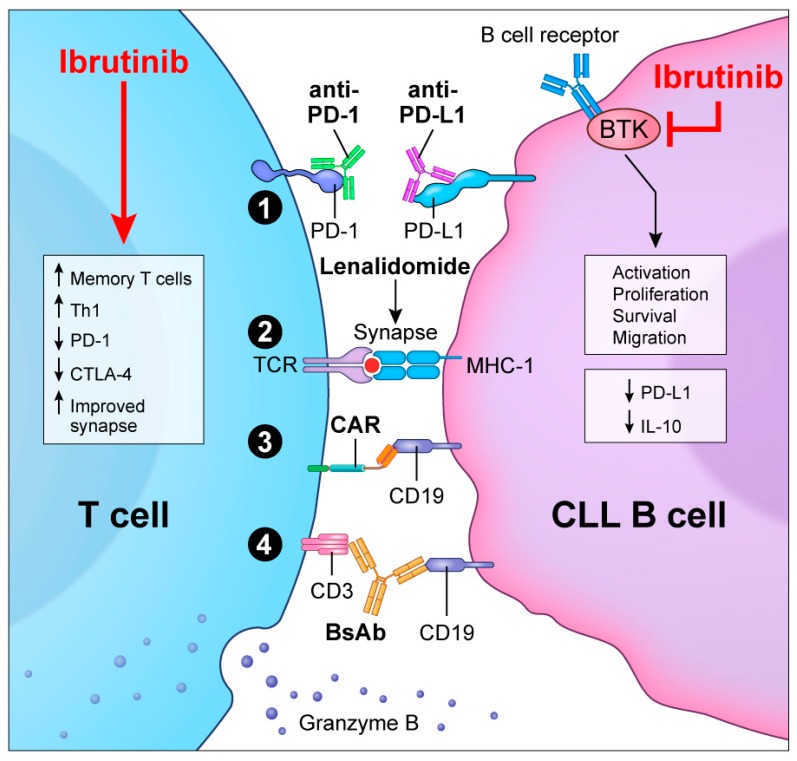
Overview of the possible combination strategies of BTKi with T-cell direct immunotherapy. As shown on the right, by targeting BTK, Ibrutinib inhibits B-cell activation, proliferation, survival pathways and the expression of immunoregulatory molecules such as PD-L1 and IL-10. In T cells, ibrutinib improves their function. The combination of ibrutinib with T-cell directed immunotherapeutic strategies such as (1) PD-1/PD-L1 blockade by antibodies; (2) immunomodulatory agents such as lenalidomide; (3) CAR-T cells or (4) BsAb is a promising treatment approach for CLL.

Despite the success of BTKi in the treatment of CLL, there remains a need for adjunct treatments capable of deepening response or overcoming drug resistance. T-cell-directed immunotherapies have demonstrated efficacy in solid tumors and some hematologic malignancies. However, disease-associated T-cell defects have impaired the activity of immunotherapies in CLL. Improved T-cell function following ibrutinib therapy provides the rationale to combine BTKi with immunotherapy (Figure 2).

### 3.1. Combining BTK Inhibition with Autologous T-Cell Therapies

#### 3.1.1. BTKi + Chimeric Antigen Receptor (CAR) T Cells

Chimeric antigen receptor (CAR) T cells are genetically modified to express chimeric receptors that bind antigens independent of the major histocompatibility complex. In CLL, the most studied CAR-T cells developed to date are directed against CD19.

The first to report of the efficacy of anti-CD19 CAR-T cells against CLL was in 2011 [64]. Porter reported a durable remission in a third of R/R CLL patients with second-generation anti-CD19 CAR-T cells [65]. Several clinical trials assessed the efficacy of CAR-T cells in CLL, showing a lower overall response rate (ORR) in R/R CLL (ORR 70%) than in R/R acute lymphocytic leukemia (ALL) patients (ORR 81%) [66].

To improve outcomes in CLL patients receiving CAR-T cells, pre-clinical and clinical studies have investigated the effect of ibrutinib treatment on CAR-T cell efficacy. In patients with CLL receiving ibrutinib, expression of inhibitory receptors PD-1, and CD160, on T cells and immunosuppressive molecule CD200 on CLL decreased, and their expression levels were negatively correlated with the proliferative capacity of CAR-T cells during ex vivo expansion. Coadministration of ibrutinib and CAR-T cells in MCL, ALL and CLL human xenograft models improved CAR T-cell engraftment, tumor clearance and survival [67,68].

Prior treatment or coadministration of ibrutinib with CAR-T cell therapy was shown to be effective with a manageable toxicity profile in CLL patients. In patients refractory to ibrutinib, the ORR was 74% and minimal residual disease (MRD) negativity was achieved in 88% of evaluable patients at four weeks after CAR-T cell infusion [69]. When CAR-T cells were administered as consolidation therapy for patients who did not achieve a complete response after at least six months of ibrutinib, a higher rate of sustained responses (CR rate of 43%) were observed compared to previous studies with CAR-T cells in ibrutinib-naïve patients (CR rates of 21–29%) [70]. In a subsequent study of CAR-T cells, ibrutinib coadministration improved response and attenuated grade ≥ 3 cytokine release syndrome (CRS) compared to CAR-T cells alone [71]. A clinical study investigating the combination of CAR-T cells and ibrutinib in CLL, small lymphocytic leukemia (SLL) and DLBCL is ongoing (NCT03960840).

#### 3.1.2. BTKi + Bispecific Antibodies (BsAbs)

BsAbs are engineered antibodies that simultaneously recognize two different antigens or epitopes expressed by effector immune cells such as T cells and target cells. In contrast to CAR-T cells, this T-cell redirection strategy is available “off-the-shelf” without the personalized manufacturing requirements of cellular therapies [72,73]. BsAbs recruit endogenous T cells to form cytolytic synapses with B cells resulting in T-cell activation, proliferation and T-cell-induced cancer cell lysis [74]. Bispecific T-cell engager (BiTE) is a type of BsAb formed by the fusion of single chain antibodies carrying one binding site for T-cell specific marker CD3 and CD19, a B-cell specific marker. Blinatumomab, a CD19/CD3 BiTE, has shown in vitro activity against CLL cells [75] and clinical efficacy in R/R ALL and non-Hodgkin lymphoma (NHL) [74,76]. A major limitation of blinatumomab is its short half-life (2.1-h) requiring continuous intravenous infusion.

To improve the ease of administration, alternative antibody formats with longer half-lives have been developed. A novel CD19/CD3 BsAb in a 100 kDa single chain-Fv Fc format (CD19/CD3-scFv-Fc) with a half-life of approximately seven days resulted in apoptosis of primary CLL cells in vitro and near complete elimination of tumor cells in a patient-derived mouse xenograft model [77]. Interestingly, T cells from ibrutinib-treated patients expanded more rapidly and exerted more cytotoxic activity than T cells from treatment-naïve CLL patients [77].

Bispecific antibodies carrying one binding site for CD20 or receptor tyrosine kinase-like orphan receptor 1 (ROR1) are also under investigation. Ibrutinib enhanced the immune synapse formation and increased the efficacy of ROR1/CD3 BsAb [78]. Therefore, coadministration of BsAbs with BTKi could enhance the activity of BsAbs. Two CD20/CD3 BsAbs, REGN1979 and mosunetuzumab, have shown promising preliminary results in patients with R/R CLL and NHL and (NCT02290951; NCT02500407).

### 3.2. Combining BTK Inhibitors with Immunomodulatory Drugs

Lenalidomide is an immunomodulatory drug (IMiD) with activity in CLL patients [79,80]. In addition to directly inhibiting CLL proliferation, lenalidomide affects the T-cell compartment [81]. In vitro treatment with lenalidomide repaired immunologic synapses between T cells and CLL cells and downregulated PD-1 and PD-L1 expression on T cells [81]. In R/R CLL patients, treatment with single-agent lenalidomide increased T-cell proliferation and Th1 polarization [82]. To limit toxicity, lenalidomide was evaluated as maintenance therapy after chemoimmunotherapy. Two randomized clinical trials (CLL M1 and CALGB10404) showed that lenalidomide maintenance reduced disease progression [83,84]. However, three unexpected cases of B-ALL in the lenalidomide maintenance arm resulted in the termination of the CLL M1 study [83].

In a phase 1 study of ibrutinib, lenalidomide and rituximab in R/R CLL, the ORR was comparable to previous reports of either single agent ibrutinib or lenalidomide plus rituximab. Grade 4 neutropenia occurred in 67% of patients and led to treatment discontinuation in 42% [85].

### 3.3. Combining BTK Inhibitors with Checkpoint Inhibitors

It has been hypothesized that the absence of response to anti-PD-1 monotherapy in CLL could be due to the elevated expression of several inhibitory checkpoints on pseudo-exhausted T cells [54]. Ibrutinib induces PD-1 downregulation, reduces the immunosuppressive properties of CLL cells and restores T-cell function. In vitro treatment with anti-PD-L1 induced more CLL cell death in T-cell cytotoxicity assays in samples obtained from patients treated with ibrutinib compared to those treated with chemoimmunotherapy [58]. In animal models of lymphoma and solid tumors, the combination of PD-L1 blockade with ibrutinib increased the number of IFN-γ-producing, tumor-specific, central memory T cells and improved disease control in A20 lymphoma-bearing mice compared to mice treated with either ibrutinib or anti PD-L1 alone. Disease regression was also observed in tumors resistant to ibrutinib monotherapy or that did not express BTK, highlighting the effect of ibrutinib on non-tumoral immune cells [86].

Despite pre-clinical evidence supporting the combination of ibrutinib and checkpoint blockade, a phase 1/2a trial of nivolumab with ibrutinib demonstrated an ORR of 61% in patients with R/R CLL, similar to the ORR previously reported with single-agent ibrutinib [87]. In contrast, the activity of PD-1/PD-L1 inhibition in Richter transformation appears to be more promising. Anti-PD-1 monoclonal antibody alone or in combination with ibrutinib achieved an ORR of 65% and 44%, respectively, in patients with Richter transformation [53]. Ongoing clinical trials combine pembrolizumab or nivolumab with BTK inhibitors ibrutinib (NCT03204188) or acalabrutinib (NCT02362035) for CLL, MCL and other NHLs. Whether co-administration of checkpoint inhibitors with ibrutinib can restrict the clonal evolution associated with BTKi resistance remains an interesting question. The observed activity of pembrolizumab in Richter’s transformation suggests that tumor cells with a higher mutation load could be more susceptible to immune attack.

## 4. Conclusions

The development of small molecule inhibitors such as ibrutinib has revolutionized the treatment of CLL. Although ibrutinib has remarkable clinical activity, current limitations include drug resistance and off-target toxicities. In parallel, directing the immune system to target malignant cells has provided significant gains in solid tumors and some hematologic malignancies such as ALL, DLBCL and Hodgkin lymphoma. In CLL, however, disease-associated T-cell abnormalities have hampered the successful translation of immunotherapeutic approaches.

Ibrutinib reshapes the T-cell compartment in CLL towards improved antitumor function. Expansion of differentiated memory T cells and a potential shift towards Th1 polarization have been observed in treated patients. Ibrutinib also reduces expression of inhibitory receptors such as PD-1 and restores immune synapse formation between T cells and CLL cells such as decreased production of IL-10 or downregulation of inhibitory molecules like PD-L1 [57]. It remains unclear to what extent these T-cell changes are related to the inhibition of ITK or the reduction in tumor bulk and immunosuppressive mechanisms active in untreated CLL. In support of the latter, improvements in T-cell immunity have been reported with second generation BTKis, acalabrutinib and zanubrutinib, which do not inhibit ITK. Combining functional studies, immunophenotypic and new technologies, such as next generation sequencing to study the TCR repertoire, or single cell RNA sequencing, will complete insights into changes in T-cell immunity mediated by BTKis. Preliminary data from combination trials of ibrutinib and T-cell-directed immunotherapy, such as CD19 CAR-T cell therapy in R/R patients, have been promising [70,71].

These therapeutic strategies may extend beyond B-cell malignancies. Ibrutinib was shown to have activity in several pre-clinical models of solid tumors such as pancreatic cancer [88], breast cancer [89] or lung cancer [90]. Ongoing clinical trials investigate the use of ibrutinib as an immunomodulator alone or in combination with PD-1/PD-L1 inhibitors in solid malignancies [91]. With better understanding of the behavior and function of T cells upon treatment with BTKi therapy, rationale immunotherapy-based combinations approaches could provide better disease control, shorten treatment duration and mitigate treatment-related toxicities associated with extended therapy.
